# Supplementary material for: Near-optimal combination of disparity across a log-polar scaled visual field
Source: PLoS Comput Biol. 2020 Apr 10;16(4):e1007699. doi: 10.1371/journal.pcbi.1007699 (PMC7176150; doi:10.1371/journal.pcbi.1007699)
Supplement: S1 Appendix — (PDF) [file pcbi.1007699.s001.pdf]

Appendix to:

Near-optimal combination of disparity across a log-polar scaled visual field

Guido Maiello[1*], Manuela Chessa[2], Peter J. Bex[3], Fabio Solari[2]

1 Department of Experimental Psychology, Justus Liebig University Giessen, Giessen, Hesse, Germany
2 Department of Informatics, Bioengineering, Robotics and Systems Engineering, University of Genoa, Genoa, Italy.
3 Department of Psychology, Northeastern University, Boston, Massachusetts, USA

* guido_maiello@yahoo.it

Visual processing throughout the model for a uniform disparity stimulus

Fig A shows the scheme proposed in Fig 2 for a stimulus that embeds a uniform disparity of 5 pixels (12 arcmin). The stereoscopic Cartesian input images (Fig Aa) are a noisy sinusoidal vertical gratings that are mapped to the cortical domain (Fig Ab) using the log-polar transform (Eq. 3). The non-linear gain of the mapping (Eq. 6) as a function of the log-scaled retinal eccentricity ξ is shown in Fig Am. The log-polar mapping warps the input images: Cartesian uniform vertical stripes appear distorted in their spatial orientation and frequency in log-polar image space.

The cortical representation of the input images is processed by a population of V1 binocular simple cell units (Eq. 11), following the phase-shift model [1, 2]. Ac shows the output of one such V1 binocular simple cell layer tuned to $\sigma = 5.12$ pixels, $\theta = 67.5$ degrees, $\Delta\psi = 40.2$ degrees. The corresponding retinal processing (Af, obtained by applying the inverse log-polar mapping to Ac) shows the space-variant effects of the log-polar transform: a V1 unit layer tuned to a single oblique cortical orientation and single cortical receptive field size is sensitive to the retinal vertical orientation, which contributes to covers all the retinal space, in specific retinal positions, where the varying receptive field sizes match the stimulus frequency.

Quadrature pairs of binocular simple cells are combined to form the responses of V1 complex cell units (Eq. 13)., i.e. we adopt the binocular energy model [1, 3, 4]. Disparity tuning first emerges at this level: units are tuned to vector disparity orthogonal to their spatial orientation. However, the representation of visual information is distributed across the parameter space of V1 complex cells. A single cell layer, tuned to specific parameters, exhibits a tuning that is related to its parameter set only, see cortical (Ad) and retinal (Ag) representations. Thus, a single cell layer contributes to only a portion of the whole representation. Fig An shows the population response of V1 complex cells tuned to the disparity d orthogonal to their spatial orientation θ. The inset of Fig An shows the activity $E^{V1}(p, \theta, d)$ (vertical axis θ and horizontal axis d). Here,we use the same parameter sampling we employed in our simulations, thus the plots are at a coarse resolutions (i.e. $K \times N$, 5×12).

At the MT level (Eq. 14), where V1 complex cell responses are pooled across spatial and orientation domains, the tuning to the vector disparity emerges. The representation

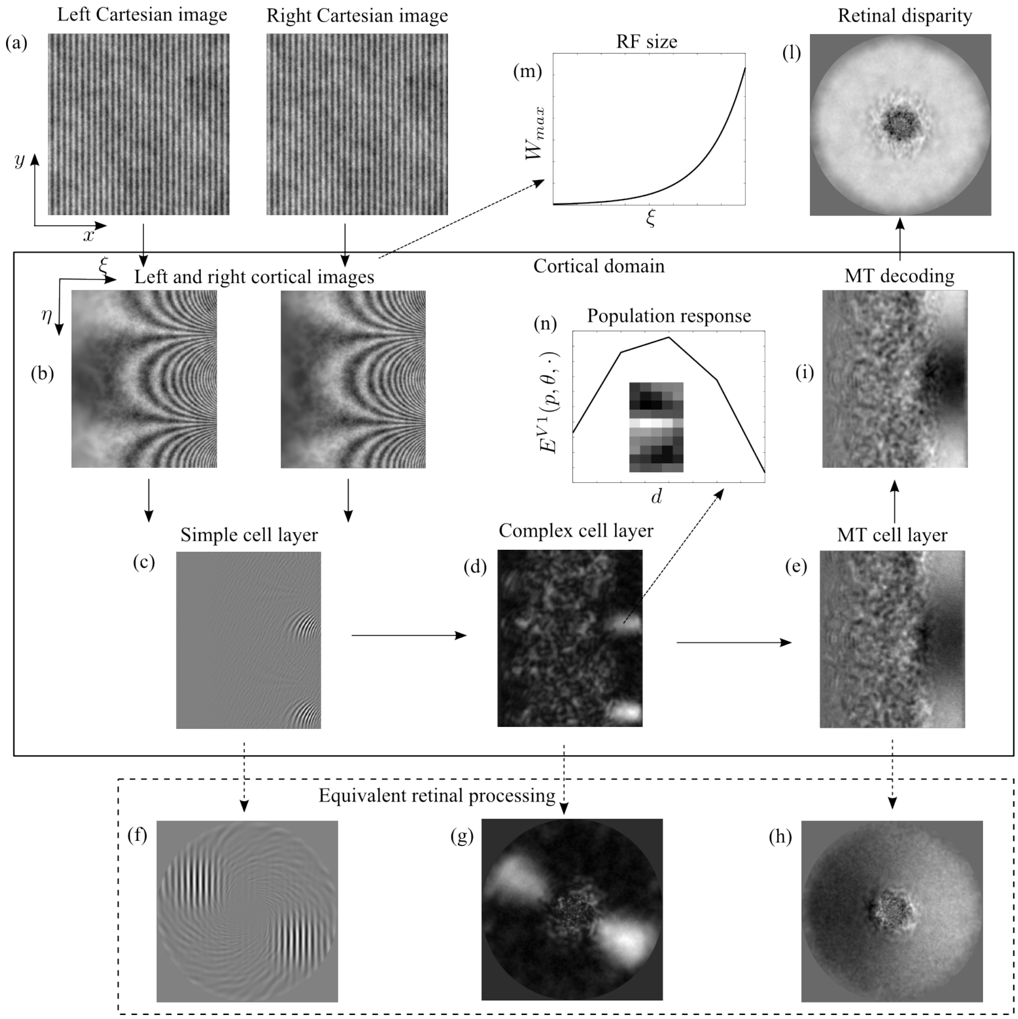

Fig A. A scheme of the proposed model and of its encoding/decoding of the visual information for a uniform disparity stimulus (see 2 for the stimuli used in our experiment). In (a) there are the left and right input stimuli: a noisy vertical sinusoidal grating that encodes a uniform disparity. Such stimuli are mapped to the corresponding cortical representation (b) through a non-linearity (m) as a function of eccentricity (Eq. 6). These cortical images are the visual afferents to V1 layers: the activity of the simple cell layer (c) is non-linearly combined to produce the complex cell layer (d) (for the sake of clarity we show an activity image for one set of tuning parameters, only). At this stage of the model the visual information is encoded in a distributed representation. Specifically, the population of complex cells (Eq. 13) show a response that is tuned to a disparity d orthogonal to their spatial orientation θ (n), moreover the inset shows the activity $E^{V1}(p, \theta, d)$. Then, by pooling afferent V1 responses the MT cell populaion activity (e) shows a tuning both to magnitude d and direction ϕ of disparity (Eq. 14). The equivalent retinal processing is shown in (f-g-h), i.e. the cortical activity is back mapped to retinal space (only for visualization purposes, this representation is not computed or utilized by the model). The MT activity is decoded (i) in order to estimate the disparity (Eq. 16). (l) shows the estimated disparity map (in the retinal domain) for a uniform disparity stimulus. This disparity is large, thus it is optimized for the model's periphery, and the estimated disparity map is thus degraded in the fovea.

of visual information is distributed across the parameter space of MT cell that directly encodes, at this level, the visual features related to the stereoscopic stimulus, i.e. magnitude d and direction ϕ. Fig Ae shows the response of an MT cell tuned to a specific cortical disparity, and Fig Ah the equivalent retinal processing, where a partial representation of the disparity information (a uniform disparity stimulus) embedded in the input images emerges. By combining these partial disparity representations we can decode (Eq. 16) the MT activity in order to obtain a full estimate of cortical disparity (Fig Ai). The estimated retinal disparity map shown in Fig Al is obtained by backwards transforming the decoded cortical activity. The input disparity value (5 pixels) matches the tuning of the model's disparity sensitivity at the periphery. Therefore, the disparity is primarily detectable in the model's periphery, and is degraded by the log-polar mapping towards the model's visual fovea.

References

1. Fleet DJ, Wagner H, Heeger DJ. Neural encoding of binocular disparity: energy models, position shifts and phase shifts. Vision research. 1996;36(12):1839–1857.

2. Qian N, Zhu Y. Physiological computation of binocular disparity. Vision research. 1997;37(13):1811–1827.

3. Ohzawa I, DeAngelis GC, Freeman RD. Stereoscopic depth discrimination in the visual cortex: neurons ideally suited as disparity detectors. Science. 1990;249(4972):1037–1041.

4. Allenmark F, Read JCA. Spatial Stereoresolution for Depth Corrugations May Be Set in Primary Visual Cortex. PLOS Computational Biology. 2011;7(8):1–14.
